# Characterization and Expression of the Gene Encoding *En*-MAPK1, an Intestinal Cell Kinase (ICK)-like Kinase Activated by the Autocrine Pheromone-Signaling Loop in the Polar Ciliate, *Euplotes nobilii*

**DOI:** 10.3390/ijms14047457

**Published:** 2013-04-03

**Authors:** Annalisa Candelori, Pierangelo Luporini, Claudio Alimenti, Adriana Vallesi

**Affiliations:** Laboratory of Eukaryotic Microbiology and Animal Biology, Department of Environmental and Natural Sciences, University of Camerino, Camerino 62032, Italy; E-Mails: annalisa.candelori@unicam.it (A.C.); piero.luporini@unicam.it (P.L.); claudio.alimenti@unicam.it (C.A.)

**Keywords:** protein kinase, signal transduction, gene structure and expression, translational frameshifting, polar ciliates

## Abstract

In the protozoan ciliate *Euplotes*, a transduction pathway resulting in a mitogenic cell growth response is activated by autocrine receptor binding of cell type-specific, water-borne signaling protein pheromones. In *Euplotes raikovi*, a marine species of temperate waters, this transduction pathway was previously shown to involve the phosphorylation of a nuclear protein kinase structurally similar to the intestinal-cell and male germ cell-associated kinases described in mammals. In *E. nobilii*, which is phylogenetically closely related to *E. raikovi* but inhabits Antarctic and Arctic waters, we have now characterized a gene encoding a structurally homologous kinase. The expression of this gene requires +1 translational frameshifting and a process of intron splicing for the production of the active protein, designated *En*-MAPK1, which contains amino acid substitutions of potential significance for cold-adaptation.

## 1. Introduction

Protein kinases are among the major regulatory components of every eukaryotic intracellular signal transduction pathway, in particular of those pathways that are activated by growth factors and cytokines deputed to control cell proliferation and differentiation. The so-called Intestinal Cell Kinases (ICKs) and Male germ cell-Associated Kinases (MAKs), originally identified from mammalian intestine and testicular germ cells, respectively, represent a large group of Ser/Thr protein kinases with a catalytic domain similar to those of the cyclin-dependent protein kinases (CDKs) and mitogen-activated protein kinases (MAPKs) [1–3]. They require a double phosphorylation of a MAPK-like Thr-Asp-Tyr motif for activation, and have a long carboxy-terminal, non-catalytic domain with putative functions in protein-protein and protein-DNA interactions [3]. Studies in particular of ICK structure and activation by growth factors suggested that these proteins operate through a signaling pathway that is distinct from the classic MAPK pathway [1,4]. Nevertheless, a substantial body of experimental evidence supports a specific correlation of ICK expression and activation with cell proliferation and cell-cycle progression, since knockdown of ICK expression in replicating intestinal epithelial cells delays cell growth rate and cell cycle progression [4]. Similarly, MAK activation has been shown to be essential for the replication of prostate epithelial cells [5,6].

In consideration of the central role that ICKs and MAKs play in the mechanisms that regulate cell proliferation, it is widely accepted that the structure and activity of these proteins are largely conserved throughout evolution. Indeed, the number of annotated ICK- and MAK-like proteins is continuously increasing in correlation with new genome sequencing from different organisms.

A protein kinase showing unmistakable structural homology to ICKs and MAKs was identified in *Euplotes raikovi*, a marine species of the ubiquitously distributed protozoan ciliate *Euplotes*, in relation to the autocrine signaling loop that promotes the vegetative growth of this microorganism [7]. This *Euplotes* kinase, designated *Er-*MAPK1, revealed a 283-amino acid amino-terminal catalytic domain with high degrees of identity (62%–65%) and similarity (82%–85%) to both ICKs and MAKs, and all the basic structural traits that are required for MAPK catalytic activity, in particular the double phosphorylation site provided by the Thr-Asp-Tyr tripeptide in the activation loop. On the other hand, the 348-amino acid carboxy-terminal domain appeared to be unique, particularly rich in glycine residues and potential sites for regulatory activities. In addition, like ICKs and MAKs, *Er-*MAPK1 was shown to reside in the nuclear apparatus, where it appears phosphorylated in growing cells which interact in autocrine fashion with their own specific (self) signaling pheromones, or dephosphorylated in cells which are induced to mate and temporarily arrest their growth by paracrine interactions with foreign (non-self) signaling pheromones [7,8].

In *E. nobilii*, a cold-loving (psychrophilic) species which has been isolated from Arctic and Antarctic waters [9,10] and is phylogenetically closely allied to the temperate-water species *E. raikovi*[11,12], we have now characterized a gene encoding a homologous kinase, designated *En* (from *E. nobilii*) MAPK1. This gene was cloned from the transcriptionally active genome of the cell somatic nucleus (macronucleus) which exclusively contains linear, gene-sized DNA molecules amplified to hundreds, or thousands of copies each carrying a single coding region flanked by non-coding regions capped with telomeres uniformly characterized by C_4_A_4_ repetitions in position 5′ and G_4_T_4_ repetitions in position 3′ [13,14]. Analysis of the *En-*MAPK1gene expression revealed that +1 transcriptional frameshifting and removal of an intron sequence are required for the synthesis of a functional *En*-MAPK1 protein which, compared to its mesophilic counterpart *Er*-MAPK1, presents amino acid substitutions that appear to be functionally correlated to cold-adaptation.

## 2. Results and Discussion

### 2.1. Identification of the *En*-MAPK1 Protein

**Figure 1:**
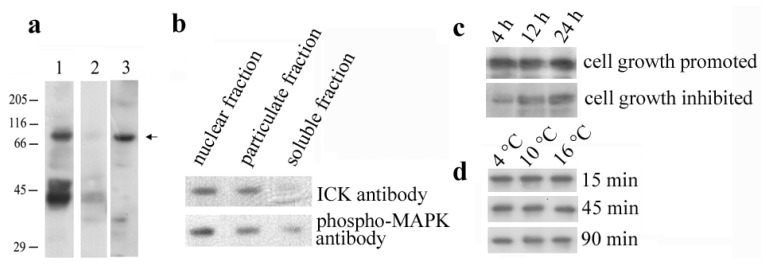
(**a**) Western blot analysis of total cell lysates probed with phospho-MAPK (lanes 1 and 2) and Intestinal Cell Kinases (ICK) antibodies (lane 3). In lane 2, cell lysates were pre-incubated with phosphatase to verify specificity of immunorecognition. The 70 kDa-band specific of *En*-MAPK1 is indicated by an arrow, while the relative positions of molecular weight markers are indicated on the left; (**b**) Sub-cellular fractions probed with phospho-MAPK and ICK antibodies, revealing major intensity of the *En*-MAPK1-specific band in the nuclear fraction; (**c**) Cells growing in the presence of their secreted (self) pheromones, or temporarily inhibited to grow by suspension with non-self pheromones for the indicated times, and then analyzed in Western blot with phospho-MAPK antibody; (**d**) Cells incubated at increasing temperatures for increasing times and analyzed by Western blot with phospho-MAPK antibody.

Two different antibodies, one directed against the MAPK double-phosphorylated Thr-Xxx-Tyr motif and the other directed against the ICK 293-amino acid catalytic domain, were first used in Western blot analysis to identify MAK and ICK homologous protein kinases in total cell lysates and sub-cellular fractions prepared from *E. nobilii* cell cultures growing suspended with their secreted (self) pheromones. Three principal protein bands of approximately 40, 50 and 70 kDa were recognized by the phospho-MAPK antibody, and the specificity of this recognition was supported by observing no antibody staining in phosphatase-treated samples (Figure 1a). However, only the 70 kDa-band was recognized also by the ICK antibody and observed to be preferentially localized in the nuclear-enriched fraction, rather than in the soluble and particulate fractions (Figure 1b). Furthermore, the phosphorylation of the 70 kDa-band appeared to be closely correlated with the cell growth stage, since the recognition of this band appreciably decreased in cells which were washed free of their secreted pheromones and suspended in the presence of non-self pheromones which, by inducing cells to mate, cause a temporary arrest of the cell growth (Figure 1c). On the other hand, no appreciable variations in the 70-kDa protein phosphorylation level were detected in cells exposed to environmental stresses caused, for example, by temperature increases (Figure 1d).

### 2.2. Molecular Cloning of the *En*-MAPK1 Coding Gene

**Figure 2:**
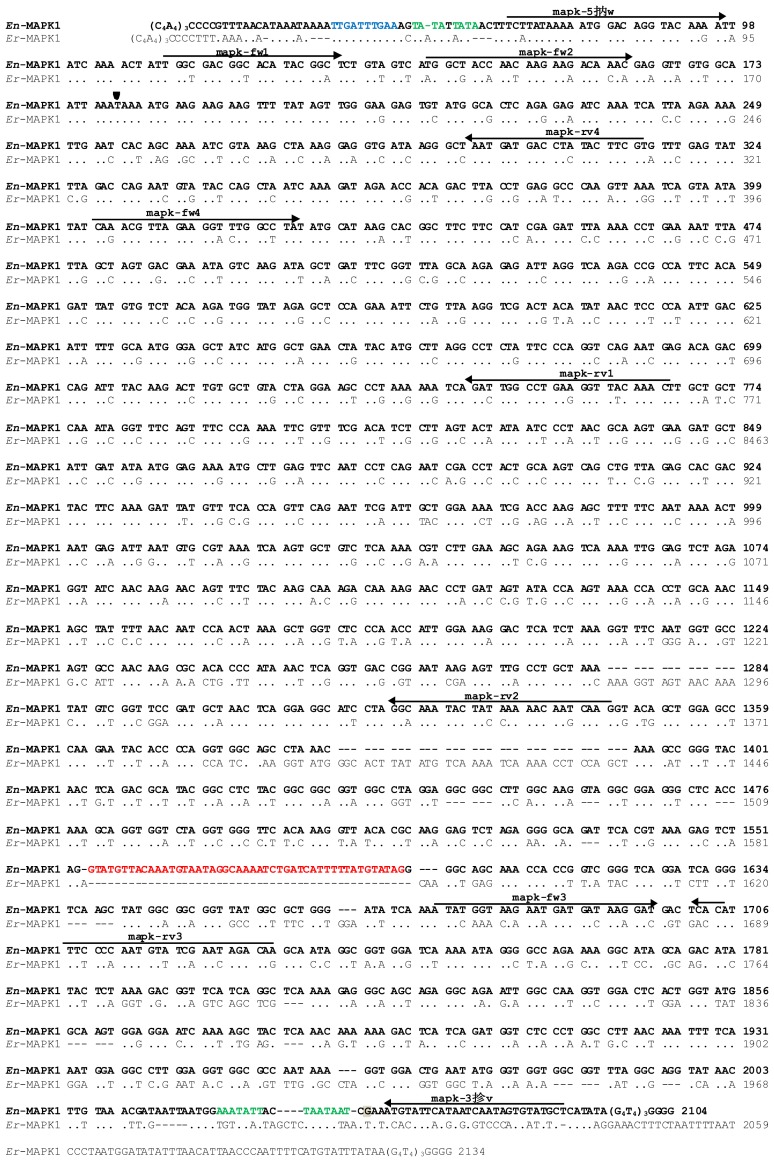
Nucleotide sequence alignment between the *En*-MAPK1 (in bold) and *Er-*MAPK1 genes. The alignment was maximized by gap insertions and dots stand for identical nucleotides. The +1 frameshifting site is indicated by an arrowhead, and the intron sequence is highlighted in red. Putative chromosome fragmentation site is in blue, while elements for the transcriptional control and polyadenylation signals are in green. The nucleotide of transcription termination is highlighted in gray. Arrows indicate positions, directions, and denominations of the PCR primers. Numbers on the right indicate the nucleotide positions.

To clone the *E. nobilii En*-MAPK1 coding gene, PCR degenerate primers (“mapk-fw1” and “mapk-rv1”, see Figure 2 and Experimental Section for primer sequences) were first designed on two segments of the known amino acid sequence of *E. raikovi Er*-MAPK1, one segment matching the ATP-binding site, Gly-Asp-Gly-Thr-Tyr-Gly-Ser, located in the catalytic amino-terminal domain, and the second segment equivalent to the sequence, Trp-Pro-Glu-Phe-Lys-Leu-Ala, that appears to be conserved in protein kinases from uni- to multi-cellular organisms. With this primer combination, a 660-bp amplification product was obtained. Other degenerate primers (“mapk-rv2” and “mapk-rv3”) were then designed on two sequence segments of the *Er-*MAPK1 carboxy-terminal domain and used in combination with a primer (“mapk-fw2”) specific for the 660-bp fragment sequence. Two additional 1200- and 1600-bp amplification products were generated by this second PCR run. The *En*-MAPK1 5′ and 3′ non-coding regions were finally obtained by a PCR amplification based on a telomere-specific primer (“Tel”) alternatively used as reverse or forward primer in combination with primers (“mapk-fw3” and “mapk-rv4”) designed on the known gene sequence.

The full-length *En*-MAPK1 gene sequence was reconstructed by overlapping all the individual sequences, and its uniqueness was confirmed by direct analysis of the products of a DNA amplification run with primers (“mapk-5′fw” and “mapk-3′rv”) equivalent to sequence stretches located near the *En*-MAPK1 5′ and 3′ telomeric ends.

### 2.3. *En*-MAPK1 Gene Structure and Expression

The *En*-MAPK1 gene (deposited at EMBL GenBank database under the accession number: KC787556) is 2104-bp long, telomeres included (Figure 2). Its 52-bp 5′ non-coding region is particularly rich in A and T and contains the motif 5′-TTGATTTGAA-3′ 17-bp downstream the 5′ telomeric repeats, which recalls the TTGAA putative consensus sequence detected in the same position in gene sequences of *E. crassus* and reputed to be involved in the organization of the sub-chromosomic (gene-sized) DNA molecules that form the cell expressed macronuclear genome [15].

The *En*-MAPK1 5′ region is similar to that of the *Er*-MAPK1 gene, most likely in relation to its activity in the regulation of transcription. In both the *En*-MAPK1 and *Er*-MAPK1 genes, a TATAA motif recalling the TATA box for the transcription initiation is present at position-18 from the ATG start codon of the open reading frame.

In the coding region, two stop codons interrupt the open reading frame. One is a TAA codon in position 180, as in the *Er*-MAPK1 gene [7], and lies within the motif AAA-TAA-A (Figure 2). It represents a “shifty stop” [16,17] implying that the *En*-MAPK1 gene undergoes +1 translational frameshifting. The second stop codon is a TAG codon in position 1735. This codon is rarely used as a stop by *Euplotes*, which preferentially utilizes TAA. To verify whether the coding region effectively ends with the TAG in position 1735, cDNAs were prepared by reverse transcription of total RNA with an oligo(dT)-linker primer; then, they were amplified with the same linker primer used in combination with a primer (“mapk-fw4”) annealing to a region of the open reading frame specific for the amino-terminal conserved catalytic domain. The sequence of a 1600-bp gene fragment obtained by this amplification revealed that the *En*-MAPK1 gene is interrupted by a 47-bp intron, particularly rich in A and T content (75%) and containing canonical 5′ GTA-TAG 3′ splice sites matching those detected in other eukaryotic organisms [18]. Consequently, the coding region of the *En*-MAPK1 gene appears to be subdivided into two exons of 1473 and 409 bp, and to end with a third inframe TAA stop codon in position 2007.

The nucleotide sequence comparison of the *En*-MAPK1 gene with its homolog *Er*-MAPK1 of *E. raikovi* shows that conservation is maximal (78% of nucleotide identity) throughout the first 1200 bp of the open reading frame. Variations almost completely reside in the third codon position, since 217 over 267 nucleotide substitutions involve this position (Figure 2).

The *En*-MAPK1 3′ region is 67-bp long and its nucleotide sequence appears to be different from that of the *Er*-MAPK1 gene. It lacks the canonical AAATAA polyadenylation signal, which is likely replaced with AAATATT, or TAATAAT motifs upstream the interruption of transcription which is coincident with the G nucleotide in position 2041.

### 2.4. *En*-MAPK1 Protein Structure

**Figure 3:**
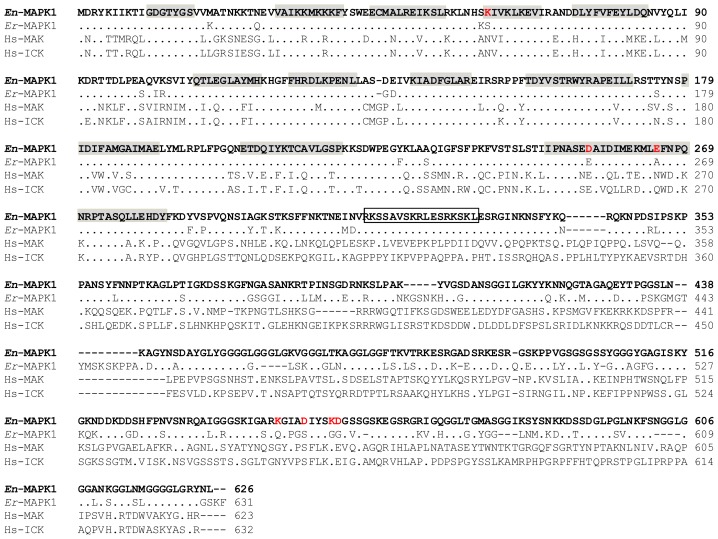
Amino acid sequence alignment of *En-*MAPK1 (in bold) with *Er-*MAPK1 (AM409185) and human MAK (NP_005897) and ICK (NP_055735). The alignment was maximized by gap insertions and dots indicate identical residues. In *En-*MAPK1 sequence, the catalytic sub-domains are highlighted in gray, while the amino acid substitutions internal to these sub-domains and within the carboxy-terminal tail are marked in red. The putative nuclear localization signal is boxed.

The *En-*MAPK1 protein includes a 283-amino acid amino-terminal domain containing all the catalytic sub-domains for the kinase activity and a 343-amino acid carboxy-terminal domain particularly rich in Gly, Ser, and basic residues. Sequences alignment of *En-*MAPK1 with *Er*-MAPK1 (Figure 3) shows that the percentages of sequence identity and similarity between the two proteins are 78% and 84%, respectively. Fourteen amino acid substitutions distinguish the *En-*MAPK1 amino-terminal domain from that of *Er*-MAPK1, and at least three of them (*i.e.*, Ser61/Lys, Glu255/Asp and Ala265/Glu) appear to be functionally important, lying in close correspondence of two different catalytic sub-domains. In the carboxyl-terminal domain, a putative bipartite nuclear localization signal represented by the sequence, Arg_312_-Lys-Ser-Ser-Ala-Val-Ser-Lys-Arg-Leu-Glu- Ser-Arg-Lys-Ser-Lys-Leu_328_, is identical in the two proteins implying a conserved function. However, *En*-MAPK1 lacks two *Er*-MAPK1-specific sequences (*i.e.*, Lys_401_-Lys_406_, and Gly_442_-Ala_452_), whilst showing an amplification of Gly-rich segments and replacing the *Er*-MAPK1 residues Gln_557_, Ser_561_, Gly_565_ and Gly_566_ all with charged residues, *i.e.*, Lys_542_, Asp_546_, Lys_550_ and Asp_551_. An enhanced exposure of charged side chains and the extension of Gly-residue repetitions likely represent aspects of *En*-MAPK1 cold-adaptation, both being able, at least in principle, to enhance the flexibility of the *En*-MAPK1 carboxy-terminal polypeptide segment.

With regard to the *En-*MAPK1 structural comparison with human ICK and MAK, sequence identities and similarities are pervasive throughout the amino-terminal catalytic domain, but completely extraneous to the carboxyl-terminal domain (Figure 3). More significant amino acid variations are comprised between Ser_242_ and Asn_270_ (*En*-MAPK1 numbering), with the two *En*-MAPK1 Ser_242_ and Ser_246_ which are replaced in human ICK and MAK with Pro_243_ and Lys_247_, and the *En*-MAPK1 short polar motif Asn_267_-Pro- Gln-Asn_270_ which is replaced with the markedly charged sequence, Asp_268_-Pro-Lys-Lys_271_.

## 3. Experimental Section

### 3.1. Cells

The Antarctic *E. nobilii* strain Far [10] provided the experimental material. It was grown in a cold-room, at 4–6 °C, under a cycle of 8 h of mild illumination and 16 h of darkness, using natural seawater (salinity, 30‰–33‰; pH, 8.1–8.2) and the green alga *Dunaniella tertiolecta* as nutrient. Cultures were deprived of food for 2–3 days and suspended in natural sea water at a density of about 10^4^ cells/mL before being used in the experiments.

### 3.2. Western Blot Analysis

Total cell lysates were prepared from cultures harvested by centrifugation, lysed in buffer (10 mM Tris-HCl, pH 7.4, 5 mM MgCl_2_, 1 mM EDTA, 0.25% sucrose, 0.5% Triton X-100) containing protease and phosphatase inhibitors, 1 mM phenylmethanesulfonyl fluoride, 1 mM sodium orthovanadate, 5 mM β-glycerophosphate and 50 mM sodium fluoride, and sonicated for 3 s. The nuclear-enriched fraction was separated by centrifugation at 800 × *g* for 2 min at 4 °C. The soluble and particulate fractions were then obtained from the supernatant by centrifugation at 17,000× *g*, for 15 min at 4 °C. Each fraction was finally suspended in 2× Laemmli sample buffer and boiled.

Cell lysates and sub-cellular fractions were separated on 10% SDS-polyacrylamide gels (by loading aliquots corresponding approximately to 5 × 10^4^ cells/lane), and separated proteins transferred onto PVDF membranes, as described previously [7]. Membranes were incubated, at 4 °C overnight, with the phospho-MAPK antibody (commercial name “phospho-p44/42 MAP kinase antibody”, Cell Signaling Technology Inc., Danvers, MA, USA) and the ICK antibody (commercial name “anti-ICK antibody”, Abnova Corporation, Taipei City, Taiwan) at a dilution of 1:1000 in TBS containing 0.1% non-fat dried milk. After washing with TBS and 0.1% Tween-20, blots were incubated, at 37 °C for 1 h, with HRP-conjugated secondary antibodies at 1:5000 dilution, and stained by enhanced chemiluminescence (ECL) (GE Healthcare, Life Sciences, Little Chalfont, UK).

The specificity of phospho-MAPK antibody recognition was verified in cells which were lysed in phosphatase buffer (50 mM Tris-HCl, pH 7.9, 10 mM MgCl_2_, 100 mM NaCl, 0.5% Triton X-100) containing protease inhibitors, and incubated with 20 units of calf intestinal phosphatase (New England Biolabs, Beverly, MA, USA) for 30 min, at 25 °C, before being processed for SDS-PAGE analysis.

### 3.3. DNA and RNA Purification, and cDNA Synthesis

DNA was prepared from cells lysed by overnight incubation in one volume of NDS buffer (500 mM EDTA, 1% SDS, 10 mM Tris/HCl, pH 9.5), containing 200 μg/mL of proteinase K, at 50 °C, according to standard protocols [7]. Total RNA was extracted from cells using the TRIzol reagent (Invitrogen, Life Technologies Corporation, Carlsbad, CA, USA) as elsewhere described [19]. For single-stranded cDNA synthesis, 5 μg of the total RNA were incubated with an oligo(dT)-linker primer and reverse transcribed with the Maxima reverse transcriptase (Fermentas International Inc., Thermo Fisher Scientific Inc., Waltham, MA, USA) in the presence of 40U RiboLock (Fermentas), as recommended by the manufacturers. Aliquots (1 μg) of the resulting cDNA were directly used as templates for the amplification reactions.

### 3.4. Polymerase Chain Reactions (PCR) and Molecular Cloning

All the PCR amplifications were run in the Eppendorf Ep-gradient Mastercycler (Eppendorf AG, Hamburg, Germany), using oligonucleotides synthesized by Invitrogen (Life Technologies Corporation) as primers. Primer designations and sequences are reported in Table 1. DNA aliquots of 0.25 μg were used as template in 50 μL-reaction mixtures containing 0.5 μM of each primer, 0.2 mM dNTPs, 1 mM MgCl_2_, and 1 U of Phusion High Fidelity DNA Polymerase (Thermo Fisher Scientific). Thirty-five PCR cycles were as a rule carried out. Each cycle consisted of a 98 °C denaturation step for 10 s, a 30 s annealing step, and a 72 °C elongation step for 10 s to 90 s, depending on the length of expected product. The temperature of the annealing step varied from 55 to 63 °C, depending on the G+C content of the primers. A final incubation step, at 72 °C for 10 min, was added to the last cycle. Amplified products were purified and cloned into the pJET1.2/blunt Cloning Vector of the CloneJET PCR Cloning kit (Fermentas), following the manufacturer’s recommendations. Sequence reactions were carried out by BMR Genomics, Padua, Italy.

## 4. Conclusions

An increasing amount of genomic and phylogenetic comparative analyses provide compelling evidence that intracellular signaling pathways rely on a large conservation of functional units even among distantly related organisms [20–22]. In this context, protein kinases involved in basic transduction mechanisms regulating cell growth and differentiation deserve particular interest for their ancient origins. The *En*-MAPK1 kinase that has been characterized from *E. nobilii* and its homologous *Er*-MAPK1, previously characterized from *E. raikovi*, appear to be both structurally closely correlated with mammal ICKs and MAKs [7]. The main structural features shared by these ciliate and mammal protein kinases, all reside at level of the twelve sub-domains of the amino-terminal catalytic region, thus implying a strict conservation also of their biological functions and mechanisms of activation. On the other hand, *En*-MAPK1 and *Er*-MAPK1 show to have equally restricted their structural specificities to their carboxy-terminal domain. These specificities determine significant variations of *En*-MAPK1 and *Er*-MAPK1 not only from mammal ICKs and MAKs, but also between themselves in relation to the *En*-MAPK1 cold-adaptation.

The apparent lack of regulatory sequences in the 5′ non-coding region of the *En*-MAPK1 gene would imply that the *En*-MAPK1 protein is constitutively synthesized. However, the fact that *En*-MAPK1 gene expression requires intron removal and +1 translational frameshifting, processes that are commonly observed in the expression of *Euplotes* genes encoding nuclear proteins with enzymatic functions [17], is indicative that *En*-MAPK1 synthesis may be regulated at transcriptional and/or translational level.

## Figures and Tables

**Table 1:** Polymerase Chain Reactions (PCR) primers.

Denominations	Sequences (5′–3′)
mapk-fw1 a	GGWGAYGGTACWTAYGGWTC
mapk-fw2	GCTACCAACAAGAAGACAAACG
mapk-fw3	TATGGTAAGAATGATGATAAGGATG
mapk-fw4	CAAACGTTAGAAGGTTTGGCCTAT
mapk-5′fw	CTTATAAAAATGGACAGGTACAAAATTAT
mapk-rv1 a	AAGYTTRTAWCCYTCWGGCCA
mapk-rv2 a	CCTTGCTTRTTYTGRTARTAYTTTC
mapk-rv3 a	TGTCTRTTWGAAACRTTTGGRAARTG
mapk-rv4	CTCAAACACGAAGTATAGGTCATC
mapk-3′rv	TGAGCATACACTATTGATTATGAATACA
Tel	CCCCAAAACCCCAAAA
Oligo(dT)-linker	ACTAGTCTCGAGTTTTTTTTTTTTTTTTTT

a Degenerate primers in which W, R and Y indicate alternatives between A and T, A and G, and C and T, respectively.
